# Rack1 regulates pro-inflammatory cytokines by NF-κB in diabetic nephropathy

**DOI:** 10.1515/med-2022-0487

**Published:** 2022-05-26

**Authors:** Keqian Wu, Rui Peng, Qiuyu Mu, Yongxue Jiang, Jingshou Chen, Rui Ming, Jie Zhao, Zheng Zhang, Yan Sun

**Affiliations:** Department of Molecular Medicine and Cancer Research Center, Chongqing Medical University, Chongqing, China; Department of Bioinformatics, Chongqing Medical University, Chongqing, China

**Keywords:** diabetic nephropathy, Rack1, NF-κB, Tnf-α, Mcp-1

## Abstract

Diabetic nephropathy (DN) is one of the chronic microvascular diseases of diabetes. Studies revealed that inflammation is involved in the development of DN. However, its mechanisms are not fully clear. Here, we screened DN-related mRNAs by RNA sequencing in the renal tissues of db/db DN mice and normal control mice. The Swiss-Model, ZDOCK 3.0.2 and PyMOL 2.3.2 were applied for bioinformatics analysis. In total, we obtained 6,820 mRNAs that were dysexpressed in DN. Among them, Receptor for Activated C Kinase 1 (Rack1) was focused on for its high fold changes and high values of fragments per kilobase million (FPKM) in both two groups (FPKM >100). Moreover, Rack1 was highly expressed in DN *in vivo* and *in vitro*. Results displayed that the expressions of pro-inflammatory cytokines Mcp-1 and Tnf-α were increased when Rack1 was overexpressed in cells cultured with low glucose while the expressions of Mcp-1 and Tnf-α were decreased when Rack1 was silenced in cells cultured with high glucose. Furthermore, results showed that the established DN inflammatory factor nuclear factor NF-kappa-B (NF-κB) was regulated by Rack1 via the direct interaction between Rack1 and NF-κB subunits P50 and P65. In summary, this identified Rack1 could play an important role in the inflammation of DN via NF-κB, which can provide new insight for DN research.

## 1 Introduction

Diabetic nephropathy (DN) is one of the most serious complications of diabetes, accounting for about 1/3 of the number of people with diabetes, which poses a great threat to human health and is one of the main causes of end-stage renal disease [1,2,3,4,5]. Studies have shown that high glucose, high fat, high blood pressure, hemodynamics, oxidative stress, inflammation and other complex pathogenic factors lead to the accumulation of renal extracellular matrix (ECM), glomerular hypertrophy, glomerulus basement thickening and glomerular sclerosis in diabetic patients, involved in the development of DN. But the exact mechanism is still unclear [6,7]. In recent years, more and more studies have shown that the inflammatory response plays an important role in the occurrence and development of DN. Studies have shown that a continuous high-sugar environment can induce inflammation, resulting in the excessive production of immune cells and the secretion of inflammatory factors, which play a certain role in promoting the occurrence and development of DN [8,9,10]. And studies have found that DN patients have inflammatory cell infiltration and increased expression levels of various inflammatory mediators. This chronic inflammatory state plays an important role in the development of early DN [11]. At the same time, accumulating evidence showed that inflammation-related factors play an important role in DN, including interleukins, adhesion factors, chemokines, TNF-α, etc. [12]. The study found that MCP-1, as one of the classic chemokines, recruits many inflammatory cells to the kidney to aggravate the inflammatory reaction damage process of the kidney [13]; TNF-α induces cell apoptosis and, at the same time through cytotoxicity direct damage to the kidneys, they are important indicators of DN inflammation [14]. Therefore, the abnormal expression of inflammatory factors plays an important role in the occurrence and development of DN. However, the mechanism involved in inflammation in DN is very complex, and its specific mechanism needs further study.

Receptor for Activated C Kinase 1 (Rack1) consists of 317 amino acids with a molecular weight of 36 kDa, and is a highly homologous (42% identical sequence) with mammalian G protein β subunit, evolutionarily highly conserved protein [15], whose coding gene is located in regions 3, 5 and 3 of the long arm of human chromosome 5, including 7 introns and 8 exons, also known as q5as G protein β Subunit like 1[guanine nucleotide binding protein (G protein) subunit β- 2 samples 1, gnb2l1]. As a highly conserved protein, Rack1 can bind to various kinases and membrane receptors through its WD40 site and participate in various signaling pathways including cAMP/PKA, MAPK, PKC, etc., and in cell growth, differentiation and immune response, Rack1 plays important roles [16]. Moreover, studies have shown that Rack1 is abnormally expressed in various disease tissues. In the study of hepatocellular carcinoma, Ruan et al. found that Rack1 localized to the ribosome can promote the expression of proliferation-related proteins c-Myc and Bcl-2 by combining with PKCβ protein and participate in the development of hepatocellular carcinoma [17]. Some scholars have found that the expression level of Rack1 and cell autophagy increased in starvation-treated Drosophila [18]. Zhao et al. [19] found that Rack1 can promote the process of autophagy by MAPK phosphorylation in liver cells. In addition, studies have shown that due to the special structure of Rack1, it can be combined with a variety of signaling proteins and cytokine receptors, and the binding is cell specific, which is involved in inflammation-related Wnt/β-catenin [20], Akt [21], STAT3 [22] and other signaling pathway activation processes, so it plays different functions in different cells. The above suggests that Rack1 may play a role in DN inflammation; however, the role and mechanism of Rack1 in DN inflammation have not been reported.

In this study, we screened 6,820 DN-related mRNAs by RNA sequencing (RNA-seq) in the renal tissues of db/db DN mice and normal control mice. Among these, Rack1 was focused on because it was not only reported as a DN-related factor but also displayed high fold changes and high values of fragments per kilobase million (FPKM) in both two groups (FPKM >100) by RNA-seq. Moreover, our results showed that Rack1 was highly expressed in DN *in vivo* and *in vitro* by quantitative real-time polymerase chain reaction (qRT-PCR) and western blot, and it could regulate the expressions of pro-inflammatory cytokines Mcp-1 and Tnf-α in mesangial cells cultured with high or low glucose by qRT-PCR and enzyme-linked immunosorbent assay (ELISA). Furthermore, the established DN inflammatory molecule NF-κB was found to be regulated by Rack1 via the direct interaction between Rack1 and NF-κB subunits p50 (NF-κB1) and p65 (RelA) by bioinformatic analysis and immunoprecipitation (IP). Therefore, it suggests that Rack1 may play a role in the inflammation of DN via targeting NF-κB. The work will provide the help to search for the key molecule for the occurrence and development of DN.

## 2 Materials and methods

### 2.1 Animals

As an approved genetic model of type 2 DN, C57BL/KsJ background db/db mice had increased body weight within 4 weeks, hyperglycemia, obvious microalbuminuria and renal impairment within 8 weeks. Eight-week-old male db/db mice and homogeneous db/m normal mice were purchased from NBRI (Nanjing, China). The 12-week-old mice were killed after the measurement of blood glucose and 24 h urine albumin excretion rate and they were divided into two groups: normal group (db/m, *n* = 6) and DN group (db/db, *n* = 6). Kidney samples were collected for RNA-seq and qRT-PCR. All experimental procedures were approved by the Animal Care and Ethical Committee of Chongqing Medical University. The protocol was in accordance with institutional guidelines for Laboratory Animal Research of Chongqing Medical University.

### 2.2 RNA-seq

RNA-seq was conducted in the renal tissue of three 12-week-old db/db mice and two 12-week-old db/m mice at BGI (Shenzhen, China). The application software TopHat is marked in the UCSC database, and the application software Cufflinks comprehensively evaluates the transcript data and detects the differential expression. When the system status is “OK” and *q* < 0.05, the difference is considered meaningful. The quality of the detected transcript is represented by the number of sequencing fragments contained in the sequencing bases per thousand transcripts per million sequencing bases.

The Ensembl BioMart database in the “R” software installation package bioMart was used to convert the RefSeq mRNA into its corresponding transcript, protein and gene information. The sequencing information that was not converted during the conversion process is removed. Most of the information in the conversion process is one to one in the RefSeq database and Ensembl, but there is also a few one-to-many information in the RefSeq database and Ensembl database.

### 2.3 Construction of overexpression plasmid

The UCSC database mouse Rack1 (NM_008143) gene sequence was used as a reference, and the software Primer 5.0 was used for primer design. The restriction enzyme sequences of *EcoRV* and *BamHI* were added to the upstream and downstream of the primer sequence according to the multiple cloning sites present in the plasmid vector pcDNA3.1(+). BLAST was used to compare the heterogeneity of the designed primers. The total RNA of mouse kidney tissue was reverse transcribed into cDNA. The rapid end amplification method was used to obtain the full-length sequence of Rack1. Two microliters of the amplified product was subjected to agarose gel electrophoresis. The sequence is consistent. The samples were double digested by *BamHI* and *EcoRV* at 37°C for 1 h, ligated by DNA T4 ligase at 16°C overnight, transformed into *Escherichia coli* DH5α competent cells and taken in LB agarose medium (containing ampicillin), cultured at 37°C for 12 h. Then, a single colony was picked and shaken at a 220 rpm shaker for 12–14 h at 37°C, the plasmid was extracted, PCR was performed, the samples were double digested and the sequencing was identified. BLAST sequencing is consistent with the Rack1 gene of UCSC mice.

### 2.4 Synthesis of Rack1 siRNA

The mouse Rack1 mRNA sequence (NM_008143) published by the UCSC database was used, and the software Oligo was used to design three sequences (siRNAsiRack1-No. 1, siRack1-No. 2 and siRack1-No. 3). The specific sequences were as follows: 5′-CCACAAUGGAUGGGUAACATT-3′, 5′-GUCAUUUCAUCACCAAUUATT-3′ and 5′-GACCACUGGUGUUUACUAUTT-3′. siRNAs were synthesized by Shanghai Shengong Biotechnology Co, Ltd (Shanghai, China), and the interference efficiency was detected by qRT-PCR.

### 2.5 Cell culture

Immortalized mouse glomerular mesangial cell SV40-MES13 cell line was purchased from the Cell Bank of the Chinese Academy of Sciences (Beijing, China). Then, the cells were cultured with different glucose concentrations to simulate normal and diabetic pathologies at 37°C and 5% CO_2_ and the dulbecco’s modified eagle medium (DMEM) containing 20% fetal calf serum with a glucose concentration of 25 mmol/L was considered as high glucose to simulate the pathological condition of diabetes; the DMEM concentration with a glucose concentration of 5.5 mmol/L with 20% fetal calf serum was considered low glucose to simulate normal condition. When the degree of cell fusion reached 80%, cells were digested with 0.25% trypsin, centrifuged at 800 rpm for 5 min and collected for further usage.

### 2.6 Cell transfection

Grouped mesangial cells cultured with high and low sugars. Mesangial cells (MCs) cultured at a low concentration of glucose and mannitol were named L-MC group, and cells transfected with lipofectamine 3000 +pcDNA3.1 empty plasmid were named L-MC NC group, lipofectamine 3000 +pcDNA3.1-Rack1 transfection. The stained cells were named L-MC overRack1 group. MCs cultured with a high concentration of glucose were named H-MC group, cells transfected with lipofectamine 3000 +siRNA NC were named H-MC siNC group and cells transfected with lipofectamine 3000 +siRNA Rack1 were named H-MC siRack1 group. Then, we plated the cells according to the growth status of the cells. After the plating is completed, the transfected mesangial cells were cultured in high glucose according to the instructions of Lipofectamine™ 3000. After 4–6 h of transfection, the medium was changed to the corresponding complete medium, and the cells were cultured at 37°C and 5% CO_2_ incubator for 24 h to collect cells for further operation.

### 2.7 qRT-PCR

Forty-eight hours after the cells were transfected, the TRIzol method was used to extract the total RNA of the cells. According to the instructions of the kit, to extract the cellular RNA, the RNA was reverse transcribed into cDNA, and then, a reverse transcription reaction was performed on ice. The cDNA obtained from the reaction was stored at 4°C for short-term storage and at −20°C for long-term storage. The primer sequences obtained from the UCSC database were subjected to qRT-PCR experiments according to the procedure. Then, the relative expression of RNA was calculated using β-actin as an internal reference according to 2^−ΔΔCT^ and duplicate wells were set up each time to calculate the average value.

### 2.8 Western blot

Cells’ density reached 80% and above, RIPA lysate was mixed with 1% PMSF, and the total protein of mesangial cells was extracted by grouping on ice. After measuring the protein concentration, it was stored at 4°C. The gel was configured according to the reagent configuration ratio, and the sample was loaded at 50 μg per well. The quantitative protein was separated and quantified on 10% SDS-PAGE and transferred to a 0.45 μm PVDF membrane. Then, the sample was blocked with a 5% skim milk shaker at room temperature for 120 min, incubated the primary antibody at 4°C with a shaker overnight, washed the TBST three times at room temperature for 10 min each, incubated the secondary antibody at room temperature for 120 min on a shaker and washed the PVDF membrane three times with TBST for 10 min each. The ECL system was used to detect the intensity of the bands of western blots, and the β-actin antibody was used as a control. The gray value of protein bands was quantified by ImageJ software.

### 2.9 ELISA

The mouse Mcp-1 ELISA kit and mouse Tnf-α ELISA kit were purchased from BOSTER Biological Technology Co., Ltd (Wuhan, China), and the tests were performed according to the manufacturer’s instructions. When the cell density reached 80%, the cells to be tested were collected in a 10 mL sterilized centrifuge tube and the test medium was diluted according to the instructions. One hundred microliters of each standard sample of each concentration was taken into a row of seven wells, one well was left with only the dilution as zero wells, 100 mL of the sample to be tested was drawn into the enzyme wells in sequence and the plate membrane was added and stood still at 37°C for 90 min. The sealing film was removed, the liquid was shaken off and gently tapped on the filter paper, the zero wells were removed, 100 µL of antibody was added to each well and the sealing film was added, incubated at 37°C for 60 min and spun dry twice according to the instructions. After completion, 100 µL of TMB stop solution was added to each well in the same order as TMB. The OD value was measured with a 450 nm microplate reader, the standard curve was calibrated according to the standard, and the concentration of the sample to be tested was calculated according to the standard.

### 2.10 Prediction of Rack1-binding proteins

Genemania [23] was used to predict Rack1-interacting proteins. The Rack1 was asked for on the website and the protein that binds to Rack1 was got according to the interrelationships displayed on the page. The online model prediction website Swiss-Model (https://swissmodel.expasy.org/interactive/) was used to model the homology of Rack1 protein with P50 protein and P65 protein. Then, Rack1 protein, P50 protein and P65 protein were uploaded to the online docking site ZDOCK Server (http://zdock.umassmed.edu/) for molecular docking. Moreover, IFACE was used to calculate the potential energy, structural complementation and static electricity. The score function was used to rigidly dock the two proteins, and the docking produces 10 TOP conformations. The TOP1 conformation was used for graphical analysis. The molecular docking simulation method was used to analyze the binding mode of Rack1 protein, P50 protein and P65 protein, PyMOL 2.3.2 was used to display the three-dimensional graph of the protein-binding mode and Ligplus 2.1 was used to analyze the protein-binding mode.

### 2.11 IP

To determine whether Rack1 was associated with P50 and P65, an IP assay was performed using the IP kit (Biyuntian Biotechnology Co, Shanghai, China) and the Rack1 and IgG antibodies (Santa Cruz Biotechnology, USA), following the manufacturer’s protocol. The total protein of mesangial cells was extracted in groups on ice, and two tubes of protein samples (200 μL each) were removed to remove non-specific binding. Fifty microliters of agarose was added to both test tubes, 1 μg of Rack1 and rabbit IgG was added for IP and it was shaken slowly at 4°C overnight. After instantaneous high-speed centrifugation, the supernatant was aspirated, the PBS was used to wash the pellet five times and 20 μL of 1× SDS-PAGE electrophoresis loading buffer was used. The vortex was added to resuspend the pellet. After processing at 100°C for 5 min, a part of the sample was taken for electrophoresis. Subsequent operations were the same as the western blot.

### 2.12 Statistical analysis

The software used for data analysis was SPSS 24.0 and Graphpad8.0. The experimental data were expressed as mean ± standard deviation (*x* ± *s*). Two groups were compared using the *t*-test, and three groups and above were compared using a single factor analysis of variance. The difference was statistically significant at *p* < 0.05.

## 3 Results

### 3.1 Rack1 is highly expressed in DN *in vivo* and *in vitro*

**Figure 1:**
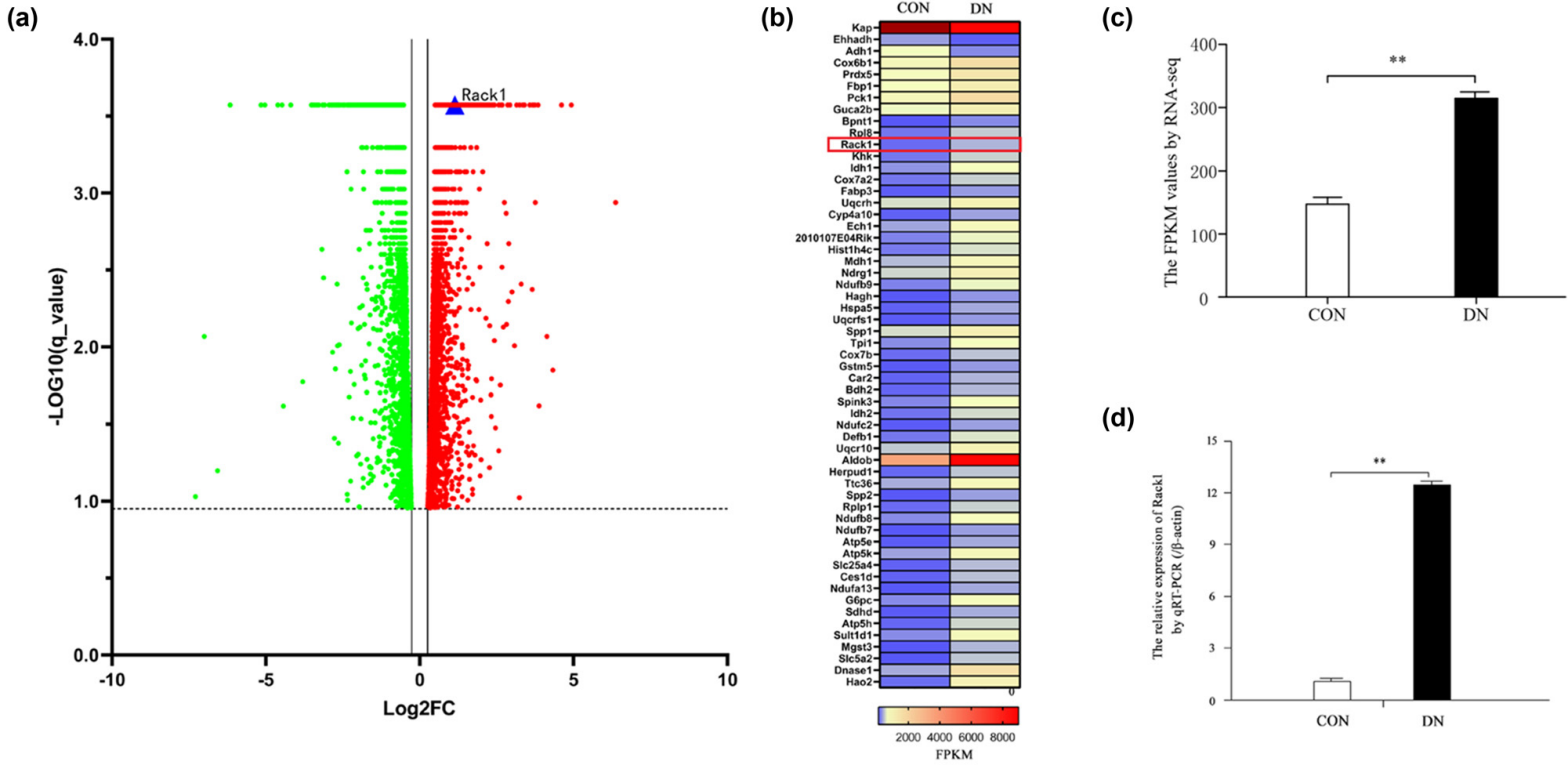
Rack1 was highly expressed in DN *in vivo* and *in vitro*. (a) The mRNA was examined by RNA-seq (*q* < 0.05) in the kidney tissues of db/db DN mice (*n* = 3 for 12 weeks) and normal control mice (*n* = 2 for 12 weeks). A total of 6,820 mRNAs were dysexpressed in the DN group, including 3,905 up-expressed and 2,915 down-expressed mRNAs (*q*-value <0.05). (b) Fifty-seven DN-related genes were found with high fold changes (|fold change| >1) and high values of FPKM in both two groups (FPKM > 100) by RNA-seq. (c) The expression of Rack1 was significantly increased in renal tissues of the DN mice group compared with that in the normal controls by RNA-seq (**p < 0.01). (d) The expression of Rack1 was significantly increased in renal tissues of the DN mice group compared with that in the normal controls by qRT-PCR. The data are representative of three independent experiments (**p < 0.01).

To explore the expression profiles of mRNAs in DN, RNA-seq was performed in the renal tissue of db/db DN mice and normal controls. The results showed that 6,820 mRNAs were dysexpressed in the DN group, including 3,905 up-expressed and 2,915 down-expressed mRNAs (*q*-value <0.05) (Figure 1a). Among these genes, 57 DN-related genes were found with high fold changes (|fold change| >1) and high values of FPKM in both groups (FPKM >100) (Figure 1b). Interestingly, Rack1 was one of them with a significant increase in the DN group (Figure 1c). To verify the result of RNA-seq, we detected the expression of Rack1 by qRT-PCR. The result showed that the expression of Rack1 was significantly increased in the DN group (Figure 1d). Therefore, it suggests that Rack1 may participate in the physiology and pathology of DN.

**Figure 2:**
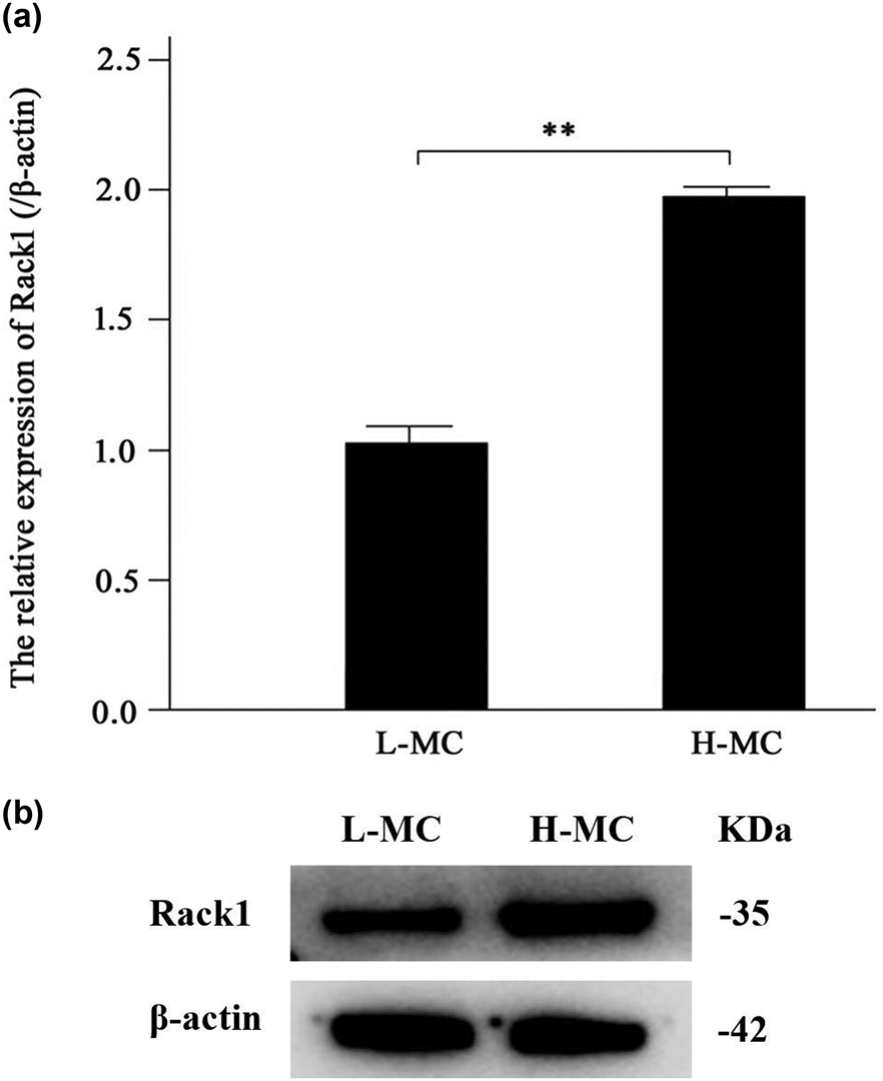
Rack1 was highly expressed in mesangial cells cultured with high glucose. (a) The expression of Rack1 was significantly increased in mesangial cells cultured with high glucose compared with that in cells of the low-glucose group by qRT-PCR. The data are representative of three independent experiments (**p < 0.01). (b) The expression of Rack1 was significantly increased in mesangial cells cultured with high glucose compared with that in cells of the low-glucose group by western blot.

Furthermore, to investigate the function of Rack1 in mesangial cells, we detected the expression of Rack1 in mesangial cells cultured with high glucose and low glucose by qRT-PCR and western blot. Our results showed that the mRNA expression of Rack1 was augmented in the high-glucose group when compared with that in the low-glucose group (Figure 2a). Moreover, data showed that the protein expression of Rack1 in the high-glucose group was higher than that in the low-glucose group (Figure 2b). This indicates that Rack1 may play a role in mesangial cells.

### 3.2 Construction of Rack1 overexpression plasmid and siRNA

**Figure 3:**
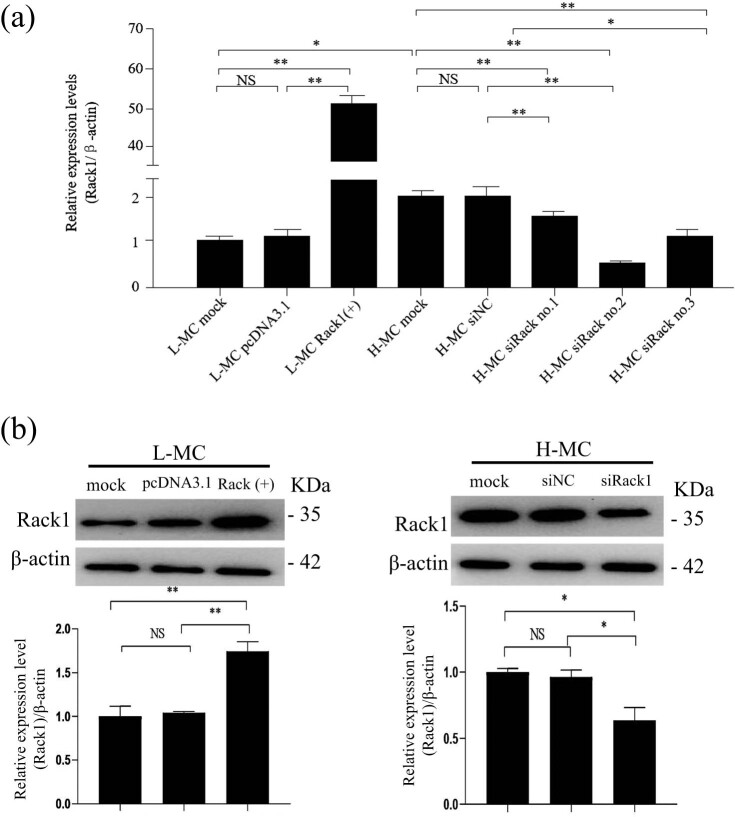
Construction of Rack1 overexpression plasmid and siRNA. Rack1 was overexpressed or silenced by Rack1(+) plasmid or Rack1 siRNAs by (a) qRT-PCR and (b) western blot. The result showed that the expression of Rack1 was significantly increased in mesangial cells transfected with Rack1 overexpression plasmid Rack1(+) in the L-MC group compared with that in cells transfected with empty plasmid pcDNA3.1 and untreated cells. Moreover, the result showed that the expression of Rack1 was decreased in mesangial cells transfected with Rack1 siRNAs (No. 2 and No. 3) in the H-MC group compared with that in cells transfected with the siRNA control and untreated cells by qRT-PCR. Data showed the effect of No. 2 was best. The data are representative of three independent experiments and presented as mean ± SD. **p < 0.01; *p < 0.05; NS, no significant.

To investigate the specific role of Rack1 in DN, we amplified the full length of Rack1 and cloned its full-length sequence into a pcDNA3.1 vector to construct a stable Rack1 over-expression plasmid, Rack1(+). The plasmid was confirmed by gel electrophoresis and sequencing after restriction enzyme digestion. Moreover, three siRNAs (siRack1-No. 1, siRack1-No. 2 and siRack1-No. 3) of Rack1 were commercially synthesized. The efficiencies of Rack1 over-expression and knockdown were detected by qRT-PCR and western blot. Data showed that Rack1 was significantly over-expressed in the cells of the L-MC group transfected with Rack1(+) plasmid and down-expressed in the cells of the H-MC group transfected with Rack1 siRNAs (Figure 3a and b). As data showed that the knockdown effect of siRack1 No. 2 was the best relative to siRack1 No. 1 and No. 3. Thus, we used siRack1No. 2 for the further experiments, referred to as siRack1 hereafter. The results indicate that Rack1 over-expression and Rack1 knockdown were successfully performed.

### 3.3 Rack1 promotes the expression of inflammatory factors of DN

**Figure 4:**
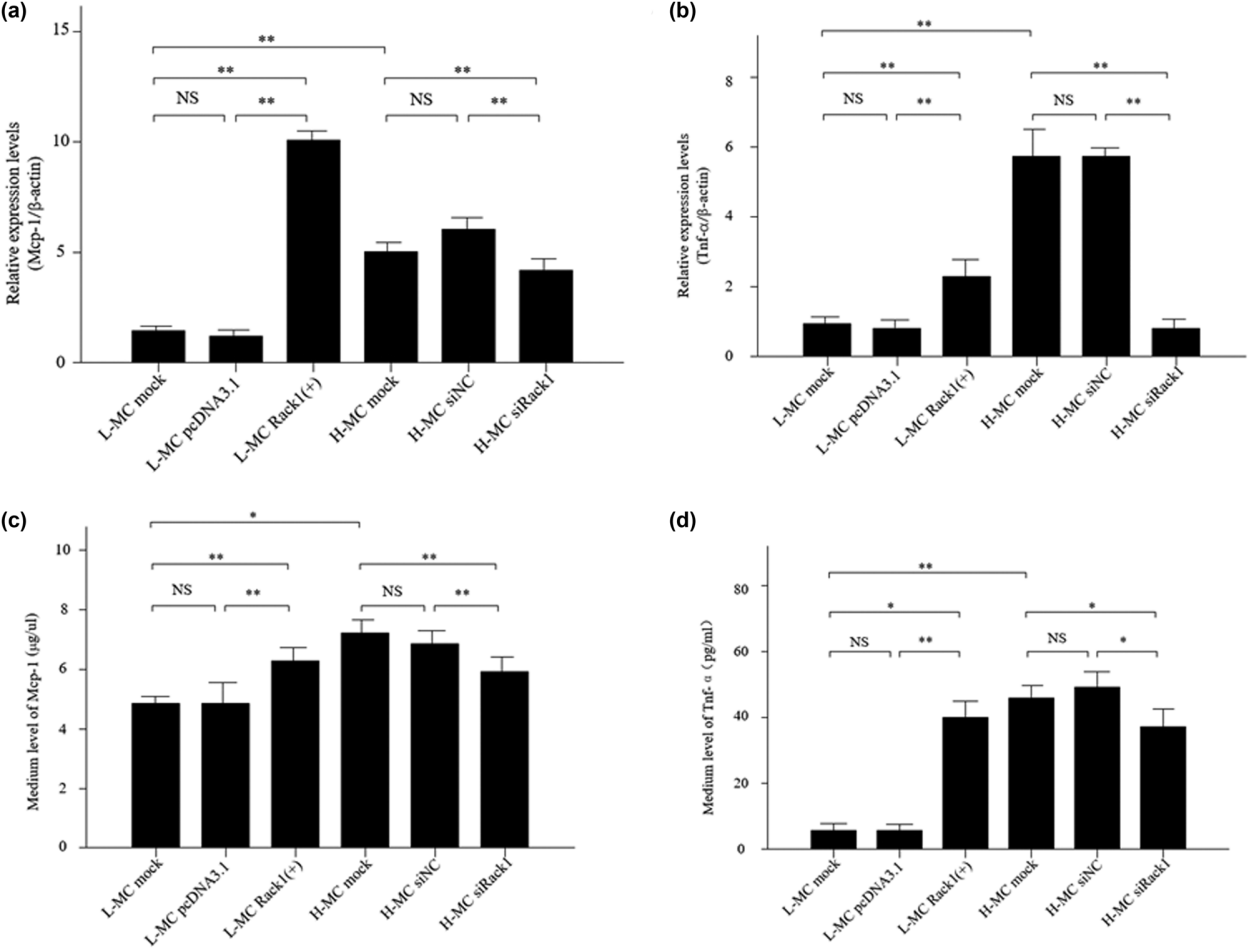
Rack1 promotes the expression of inflammatory factors in mesangial cells under high glucose conditions. (a) The expression of Mcp-1 was regulated by Rack1 by qRT-PCR. The result showed that the expression of Mcp-1 was significantly increased in mesangial cells transfected with Rack1 overexpression plasmid Rack1(+) in the L-MC group compared with that in cells transfected with empty plasmid pcDNA3.1 and untreated cells, while the expression of Mcp-1 was decreased in mesangial cells transfected with siRack1 in the H-MC group compared with that in cells transfected with the siRNA control and untreated cells. The data are representative of three independent experiments. Data are presented as mean ± SD. ^**^ *p* < 0.01; NS, not significant. (b) The expression of Tnf-α was regulated by Rack1 by qRT-PCR. The result showed that the expression of Tnf-α was significantly increased in mesangial cells transfected with Rack1 overexpression plasmid Rack1(+) in the L-MC group compared with that in cells transfected with empty plasmid pcDNA3.1 and untreated cells, while the expression of Tnf-α was decreased in mesangial cells transfected with siRack1 in the H-MC group compared with that in cells transfected with the siRNA control and untreated cells. The data are representative of three independent experiments. Data are presented as mean ± SD. ^**^ *p* < 0.01;, NS, not significant. (c) The expression of Mcp-1 was regulated by Rack1 by ELISA. The result showed that the expression of Mcp-1 was significantly increased in mesangial cells transfected with Rack1 overexpression plasmid Rack1(+) in the L-MC group compared with that in cells transfected with empty plasmid pcDNA3.1 and untreated cells, while the expression of Mcp-1 was decreased in mesangial cells transfected with siRack1 in the H-MC group compared with that in cells transfected with the siRNA control and untreated cells. The data are representative of three independent experiments. Data are presented as mean ± SD. ^*^ *p* < 0.05, ^**^ *p* < 0.01; NS, not significant. (d) The expression of Tnf-α was regulated by Rack1 by ELISA. The result showed that the expression of Tnf-α was significantly increased in mesangial cells transfected with Rack1 overexpression plasmid Rack1(+) in the L-MC group compared with that in cells transfected with empty plasmid pcDNA3.1 and untreated cells, while the expression of Tnf-α was decreased in mesangial cells transfected with siRack1 in the H-MC group compared with that in cells transfected with the siRNA control and untreated cells. The data are representative of three independent experiments. Data are presented as mean ± SD. ^*^ *p* < 0.05, ^**^ *p* < 0.01; NS, not significant.

As known, inflammation has been considered an important link in the process of DN. Then, the expressions of the pro-inflammatory cytokines Mcp-1 and Tnf-α were examined by qRT-PCR and ELISA in the mesangial cells cultured under high glucose and low glucose conditions. The results of qRT-PCR (Figure 4a and b) and ELISA (Figure 4c and d) showed that the expressions of Mcp-1 and Tnf-α were significantly higher in the high-glucose group. However, the levels of MCP-1 and Tnf-α were significantly increased when Rack1 was over-expressed in the low-glucose group, while the expressions of MCP-1 and Tnf-α were decreased when Rack1 was silenced in the high-glucose group. Therefore, these data suggest that Rack1 may regulate inflammation in MCs.

### 3.4 Rack1 regulates inflammation of DN via direct interaction with NF-κB

**Figure 5:**
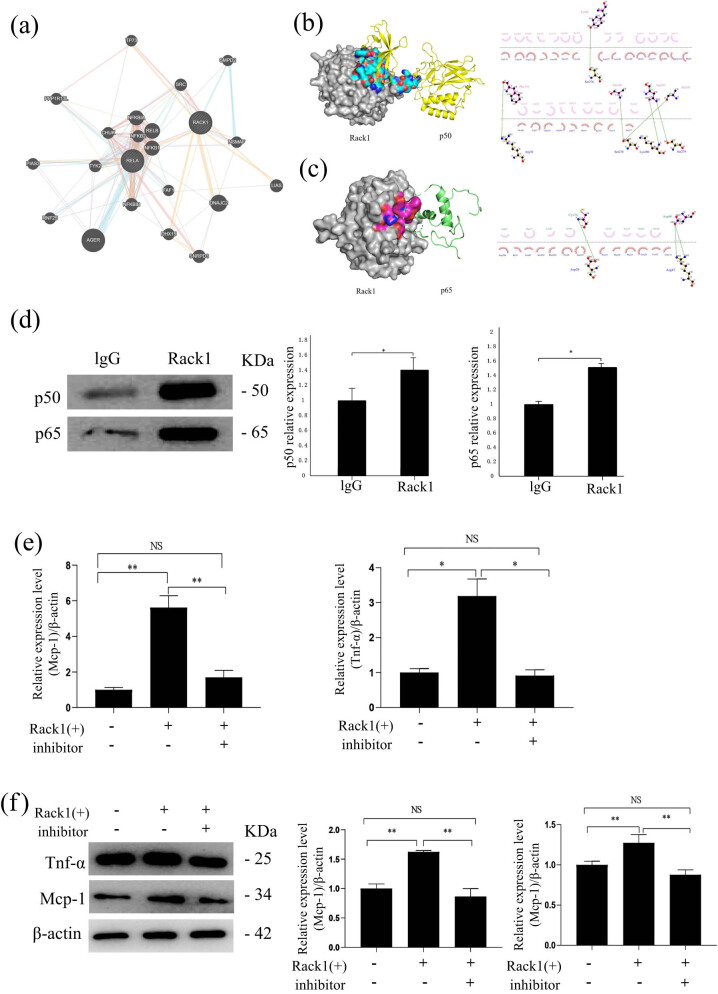
Rack1-regulated inflammation of DN via direct interacting with NF-κB. (a) Predicted Rack1 protein interaction through bioinformatics. Lines indicated possible interaction mechanisms (line color: pink lines indicated physical interactions, indicating that they were related to each other, purple lines indicated co-expression, indicating that gene expression levels were similar and are interrelated, and yellow lines indicated predicted gene). The functional relationship between the circles indicated the degree of correlation. The larger the circle was, the higher the degree of correlation was. (b) PyMOL 2.3.2 was used to display the binding pattern of Rack1 protein and P50 protein in three dimensions, and Ligplus 2.1 was used to analyze the binding pattern. Amino acid residue binding: pink indicated p50 and red indicated Rack1. The result showed that P50 was a predicted binding protein of Rack1. (c) PyMOL 2.3.2 was used to display the binding pattern of Rack1 protein and P65 protein in three dimensions, and Ligplus 2.1 was used to analyze the binding pattern. The result showed that P65 was a predicted binding protein of Rack1. Amino acid residue binding: pink indicated p65 and red indicated Rack1. (d) The combination of Rack1 and p50 by IP and quantitative analysis. The result showed that P50 was directly bound to Rack1. The data are representative of three independent experiments. Data are presented as mean ± SD. ^*^ *p* < 0.05. And the combination of Rack1 and p65 by IP and quantitative analysis. The result showed that P65 was directly bound to Rack1. (e and f) Mesangial cells were transfected with Rack1 overexpression plasmid Rack1or/and NF-kB inhibitor (CAPE) in the L-MC group. Expression of Mcp-1 and Tnf-α were determined by qRT-PCR (e) and western blot (f). These results showed that the inhibitor of NF-kB can recur that Rack1(+) increased the expression of the inflammatory factors Tnf-α and Mcp-1. The data are representative of three independent experiments. Data are presented as mean ± SD. ^*^ *p* < 0.05.

It is known that Rack1 acts as the scaffolding protein and plays role in diseases by interacting with the related proteins. Therefore, to explore the proteins related to Rack1 in DN, we used bioinformatics methods to find Rack1–protein interactions. Interestingly, two units of NF-κB (P50 and P65) were found among these predicted Rack1-related proteins; the data showed that they are interrelated for their co-expression, physical interaction, co-localization and other relationships (Figure 5a). In the study of diabetes nephropathy, NF-κB is an important inflammatory molecule, and two important constituent subunits of NF-κB have attracted our attention. Data showed that both P50 and P65 were interact with Rack1. The results of molecular docking showed that both P50 and P65 interact with Rack1, and there were multiple binding sites. According to the docking results, we found that the amino acids Ser276, Arg36, Ser278, Lys280 and Ser279 in the Rack1 protein can bind to the amino acids Tyr60, Phe310, Ala248, Asn247 and Ala245 in the P50 protein through hydrogen-bonding interaction, and the amino acid Asp29 in the Rack1 protein and Arg47 can bind to the amino acids Cys76 and Asp80 in P65 protein through hydrogen-bonding interaction (Figure 5b and c). Moreover, the result of IP displayed directly bound to P50 and P65 (Figure 5d). These results suggest that Rack1 may regulate the well-known inflammation molecule NF-κB by directly binding to P50 and P65 in DN. To verify whether Rack1 regulated inflammation of mesangial cells through modulating the NF-κB activity, we treated mesangial cells that were transfected with Rack1 overexpression plasmid or/and specific NF-κB inhibitor, caffeic acid phenethyl ester (CAPE). Expected results showed that the inhibitor of NF-kB can recure that Rack1(+) increased the expression of the inflammatory factors Tnf-α and Mcp-1 by qRT-PCR and western blot (Figure 5e and f). These results suggest that NF-κB may regulate the inflammation molecule of Rack1 in DN.

## 4 Discussion

DN is a serious diabetic microvascular complication, which is one of the main causes of end-stage renal disease. The cause of the disease is complex. It is difficult to study a single gene thoroughly to clarify its pathogenesis. In recent years, with the rise of systems biology methods and the accumulation of large amounts of high-throughput data, RNA-seq has been used to find disease-related genes [24], including DN [25,26,27], but the exactly related genes of DN are still unclear. Therefore, in this study, we screened 6,820 DN-related mRNAs in the kidney tissues of db/db DN mice and normal control mice by RNA-seq. Among them, Rack1 showed high fold change and high FPKM value in both groups (FPKM >100). In addition, our results showed that through qRT-PCR and western blot, Rack1 was highly expressed in DN both *in vivo* and *in vitro*, suggesting that Rack1 may be involved in the physiology and pathology of DN.

To study the function and possible mechanism of Rack1 in DN, we amplified the full length of Rack1 and cloned its full-length sequence into a pcDNA3.1 vector to construct a stable Rack1 overexpression plasmid Rack1(+). After restriction enzyme digestion, the plasmid was confirmed by gel electrophoresis and sequencing. In addition, three siRNAs for Rack1 (siRack1-No. 1, siRack1-No. 2 and siRack1-No. 3) were commercially synthesized. The efficiency of Rack1 overexpression and knockdown was detected by qRT-PCR. The data showed that Rack1 was significantly overexpressed in the cells of the L-MC group transfected with the Rack1(+) plasmid, while Rack1 was clearly expressed in the cells of the H-MC group transfected with Rack1 siRNA. The results of qRT-PCR showed that H-MC siRNA Rack1 No. 2 had the best silencing efficiency (*p* < 0.01), which was used in subsequent experiments and was named siRack1. Interestingly, some scholars have also studied the expression of silenced genes, and the results show that the constructed plasmids and synthetic siRNAs are more desirable in biological applications [28,29], providing broad prospects for biomedical research. The above results suggest that Rack1 overexpression plasmid and Rack1 siRNA were successfully constructed, which laid the foundation for subsequent research.

Kidney inflammation has been confirmed to cause DN and plays an important role in speeding up the progression of DN [30]. In DN patients, the level of TNF-α and MCP-1 was closely related to renal injury [31]. Upregulated TNF-α and MCP-1 caused renal tubular epithelial cells’ dysfunction, podocyte injury, mesangial cell proliferation and oxidative stress and induced mesangial cells, glomerulus and renal tubular epithelial cells to differentiate into fibroblasts, so as to enhance the production and deposition of ECM, which further enhances the progression of DN [32,33,34]. As known, a large number of studies have found that increased expression of inflammatory factors in mesangial cells can promote cell proliferation, mesangial hyperplasia, and accelerate the progression of DN disease [35,36,37]. To explore the function of Rack1 in DN inflammation, we used qRT-PCR and ELISA to identify the effect of Rack1 on inflammation in MCs cultured under high-glucose conditions. Then, the expressions of pro-inflammatory cytokines Mcp-1 and Tnf-α in MCs cultured under high- and low-glucose conditions were detected in our study. The results showed that the expressions of Mcp-1 and Tnf-α in the high-glucose group were significantly higher. However, when Rack1 was overexpressed in the low-glucose group, the levels of MCP-1 and Tnf-α were increased significantly, while when Rack1 was silenced in the high-glucose group, the expression of MCP-1 and Tnf-α was decreased. Therefore, these data suggest that Rack1 may regulate the inflammation of MCs. At the same time, studies have shown that Rack1 is wideteracted with Rack1 and there were multiple binding sites. According to the docking results, we found that the amino acids Ser276, Arg36, Ser278 and Lys280 are mainly involved in inflammation-related diseases [38,39,40], suggesting that Rack1 is involved in the occurrence and development of inflammation and is an inflammation-related factor. In summary, it suggests that Rack1 may regulate MC inflammation and promote the expression of DN inflammatory factors.

To further explore the mechanism of Rack1 in DN inflammation, we used bioinformatics methods to screen Rack1-interacting proteins. Our data indicated that many proteins were considered to be Rack1-binding proteins. Interestingly, the NF-κB subunits p50 and p65 were directly related to Rack1. The data showed that they are interrelated for their co-expression (17.38%), physical interaction (64.66%), co-localization (3.22%) and other relationships. Interestingly, we found through further research that both P50 and P65 and Ser279 in the Rack1 protein could bind to the amino acids Tyr60, Phe310, Ala248, Asn247 and Ala245 in the P50 protein through hydrogen-bonding interaction and the amino acid Asp29 in the Rack1 protein. With Arg47, it could bind to the amino acids Cys76 and Asp80 in P65 protein through hydrogen-bonding interaction. In addition, the IP results indicated that Rack1 was bound to both p50 and p65. Therefore, these results indicate that Rack1 may promote the development of DN by interacting with NF-κB subunits p50 and p65.

NF-κB is a widely existing protein molecule with multidirectional regulation, which plays an important role in the process of cell signal transmission and gene expression induction [41,42]. NF-κB, as a transcription factor of the Rel protein family, includes five subunits of p50 (NF-κB1), p65 (RelA), p52 (NF-κB2), RelB and c-Rel in mammals. There is a Rel homology region (Rel homelogymain, RHD), which can form heterologous or homodimers and participate in the inflammatory response, body immunity, cell differentiation and other processes. Among them, the dimer composed of p50 and p65 is the earliest NF-κB. Studies have shown that changes in the related regulatory pathways caused by the activation of NF-κB lead to the occurrence of inflammation and nuclear translocation [43]. As we all know, in hyperglycemia, the activation of NF-κB has been regarded as a key step in the pathogenesis of DN [10]. At the same time, the previous study of the research group found that the activation of NF-κB in mesangial cells cultured *in vitro* can promote the secretion of pro-inflammatory factors, accelerate the proliferation of mesangial cells and further cause the progression of DN [44]. The above suggests that NF-κB may play an important role in the inflammation of DN mesangial cells. In addition, studies have shown that a high glucose environment can induce the activation of NF-κB [45,46] and promote the expression of inflammation-related factors Tnf-α, Mcp-1, etc. Combined this with our results, it suggests that Rack1 may participate in DN inflammation via targeting NF-κB.

## 5 Conclusion

In summary, the present study identified the expression profiles of mRNAs in DN. Moreover, our results revealed that Rack1 may play a critical role in the inflammation of mesangial cells in DN. Furthermore, we demonstrated that Rack1 can directly interact with NF-κB subunits. In addition, we found that Rack1 could regulate the NF-κB expression in DN. Therefore, the modulation of Rack1 may provide an intriguing approach for tackling inflammation of renal mesangial cells in DN. It may provide a reliable theoretical and experimental basis for the elaboration of the pathogenesis of DN.

## References

[1] Atkins RC, Zimmet P. Diabetic kidney disease: act now or pay later. Nat Rev Nephrol. 2010 Mar;6(3):134–6. 10.1038/nrneph.2010.10. 20186229

[2] Bhattacharjee N, Barma S, Konwar N, Dewanjee S, Manna P. Mechanistic insight of diabetic nephropathy and its pharmacotherapeutic targets: an update. Eur J Pharmacol. 2016 Nov 15;791:8–24. 10.1016/j.ejphar.2016.08.022. 27568833

[3] Jha V, Garcia-Garcia G, Iseki K, Li Z, Naicker S, Plattner B, et al. Chronic kidney disease: global dimension and perspectives. Lancet. 2013 Jul 20;382(9888):260–72. 10.1016/S0140-6736(13)60687-X. 23727169

[4] Manna P, Kalita J. Beneficial role of vitamin K supplementation on insulin sensitivity, glucose metabolism, and the reduced risk of type 2 diabetes: A review. Nutrition. Jul-Aug 2016;32(7–8):732–9. 10.1016/j.nut.2016.01.011. 27133809

[5] Kelly KJ, Dominguez JH. Rapid progression of diabetic nephropathy is linked to inflammation and episodes of acute renal failure. Am J Nephrol. 2010;32(5):469–75. 10.1159/000320749. 20956853

[6] Kolset SO, Reinholt FP, Jenssen T. Diabetic nephropathy and extracellular matrix. J Histochem Cytochem. 2012 Dec;60(12):976–86. 10.1369/0022155412465073. PMC3527883 23103723

[7] Mason RM, Wahab NA. Extracellular matrix metabolism in diabetic nephropathy. J Am Soc Nephrol. 2003 May;14(5):1358–73. 10.1097/01.asn.0000065640.77499.d7. 12707406

[8] Sun Y, Peng R, Peng H, Liu H, Wen L, Wu T, et al. miR-451 suppresses the NF-kappaB-mediated proinflammatory molecules expression through inhibiting LMP7 in diabetic nephropathy. Mol Cell Endocrinol. 2016 Sep 15;433:75–86. 10.1016/j.mce.2016.06.004. 27264074

[9] Kanasaki K, Taduri G, Koya D. Diabetic nephropathy: the role of inflammation in fibroblast activation and kidney fibrosis. Front Endocrinol (Lausanne). 2013 Feb 6;4:7. 10.3389/fendo.2013.00007. PMC3565176 23390421

[10] Yi H, Peng R, Zhang LY, Sun Y, Peng HM, Liu HD, et al. LincRNA-Gm4419 knockdown ameliorates NF-kappaB/NLRP3 inflammasome-mediated inflammation in diabetic nephropathy. Cell Death Dis. 2017 Feb 2;8(2):e2583. 10.1038/cddis.2016.451. PMC5386454 28151474

[11] Sun YM, Su Y, Li J, Wang LF. Recent advances in understanding the biochemical and molecular mechanism of diabetic nephropathy. Biochem Biophys Res Commun. 2013 Apr 19;433(4):359–61. 10.1016/j.bbrc.2013.02.120. 23541575

[12] Pichler R, Afkarian M, Dieter BP, Tuttle KR. Immunity and inflammation in diabetic kidney disease: translating mechanisms to biomarkers and treatment targets. Am J Physiol Ren Physiol. 2017 Apr 1;312(4):F716–31. 10.1152/ajprenal.00314.2016. PMC6109808 27558558

[13] Haller H, Bertram A, Nadrowitz F, Menne J. Monocyte chemoattractant protein-1 and the kidney. Curr Opin Nephrol Hypertens. 2016 Jan;25(1):42–9. 10.1097/MNH.0000000000000186. 26625862

[14] Duran-Salgado MB, Rubio-Guerra AF. Diabetic nephropathy and inflammation. World J Diabetes. 2014 Jun 15;5(3):393–8. 10.4239/wjd.v5.i3.393. PMC4058744 24936261

[15] Ron D, Chen CH, Caldwell J, Jamieson L, Orr E, Mochly-Rosen D. Cloning of an intracellular receptor for protein kinase C: a homolog of the beta subunit of G proteins. Proc Natl Acad Sci U S A. 1994 Feb 1;91(3):839–43. 10.1073/pnas.91.3.839. PMC521407 8302854

[16] Adams DR, Ron D, Kiely PA. RACK1, A multifaceted scaffolding protein: Structure and function. Cell Commun Signal. 2011 Oct 6;9:22. 10.1186/1478-811X-9-22. PMC3195729 21978545

[17] Ruan Y, Sun L, Hao Y, Wang L, Xu J, Zhang W, et al. Ribosomal RACK1 promotes chemoresistance and growth in human hepatocellular carcinoma. J Clin Invest. 2012 Jul;122(7):2554–66. 10.1172/JCI58488. PMC3386807 22653060

[18] Erdi B, Nagy P, Zvara A, Varga A, Pircs K, Ménesi D, et al. Loss of the starvation-induced gene Rack1 leads to glycogen deficiency and impaired autophagic responses in Drosophila. Autophagy. 2012 Jul 1;8(7):1124–35. 10.4161/auto.20069. PMC3429548 22562043

[19] Zhao Y, Wang Q, Qiu G, Zhou S, Jing Z, Wang J, et al. RACK1 promotes autophagy by enhancing the Atg14L-Beclin 1-Vps34-Vps15 complex formation upon phosphorylation by AMPK. Cell Rep. 2015 Nov 17;13(7):1407–17. 10.1016/j.celrep.2015.10.011. 26549445

[20] Deng YZ, Yao F, Li JJ, Mao ZF, Hu PT, Long LY, et al. RACK1 suppresses gastric tumorigenesis by stabilizing the beta-catenin destruction complex. Gastroenterology. 2012 Apr;142(4):812–23.e15. 10.1053/j.gastro.2011.12.046. 22240482

[21] Wang F, Yamauchi M, Muramatsu M, Osawa T, Tsuchida R, Shibuya M. RACK1 regulates VEGF/Flt1-mediated cell migration via activation of a PI3K/Akt pathway. J Biol Chem. 2011 Mar 18;286(11):9097–106. 10.1074/jbc.M110.165605. PMC3058986 21212275

[22] Zhang W, Zong CS, Hermanto U, Lopez-Bergami P, Ronai Z, Wang LH. RACK1 recruits STAT3 specifically to insulin and insulin-like growth factor 1 receptors for activation, which is important for regulating anchorage-independent growth. Mol Cell Biol. 2006 Jan;26(2):413–24. 10.1128/MCB.26.2.413-424.2006. PMC1346890 16382134

[23] Montojo J, Zuberi K, Rodriguez H, Kazi F, Wright G, Donaldson SL, et al. GeneMANIA cytoscape plugin: fast gene function predictions on the desktop. Bioinformatics. 2010 Nov 15;26(22):2927–8. 10.1093/bioinformatics/btq562. PMC2971582 20926419

[24] Scimone C, Donato L, Alafaci C, Granata F, Rinaldi C, Longo M, et al. High-throughput sequencing to detect novel likely gene-disrupting variants in pathogenesis of sporadic brain arteriovenous malformations. Front Genet. 2020 Feb 28;11:146. 10.3389/fgene.2020.00146. PMC7059193 32184807

[25] Zhang L, Zhou Y, Zhou F, Yu X, Liu J, Liu Y, et al. Altered expression of long noncoding and messenger RNAs in diabetic nephropathy following treatment with rosiglitazone. Biomed Res Int. 2020 Jan 14;2020:1360843. 10.1155/2020/1360843. PMC6983290 32025515

[26] Li W, Sargsyan D, Wu R, Li S, Wang L, Cheng D, et al. DNA methylome and transcriptome alterations in high glucose-induced diabetic nephropathy cellular model and identification of novel targets for treatment by tanshinone IIA. Chem Res Toxicol. 2019 Oct 21;32(10):1977–88. 10.1021/acs.chemrestox.9b00117. PMC8182679 31525975

[27] Li S, Li W, Wu R, Yin R, Sargsyan D, Raskin I, et al. Epigenome and transcriptome study of moringa isothiocyanate in mouse kidney mesangial cells induced by high glucose, a potential model for diabetic-induced nephropathy. AAPS J. 2019 Dec 5;22(1):8. 10.1208/s12248-019-0393-z. 31807911

[28] Sharma R, Zhang I, Shiao TC, Pavan GM, Maysinger D, Roy R. Low generation polyamine dendrimers bearing flexible tetraethylene glycol as nanocarriers for plasmids and siRNA. Nanoscale. 2016 Mar 7;8(9):5106–19. 10.1039/c5nr06757j. 26868181

[29] Shan X, Xu T, Liu Z, Hu X, Zhang YD, Wang B. Safety and toxicology of the intravenous administration of Ang2siRNA plasmid chitosan magnetic nanoparticles. Mol Med Rep. 2017 Feb;15(2):736–42. 10.3892/mmr.2016.6090. PMC5364838 28035391

[30] Tang SC, Yiu WH. Innate immunity in diabetic kidney disease. Nat Rev Nephrol. 2020 Apr;16(4):206–22. 10.1038/s41581-019-0234-4. 31942046

[31] Liu C, Zhao S, Zhu C, Gao Q, Bai J, Si J, et al. Ergosterol ameliorates renal inflammatory responses in mice model of diabetic nephropathy. Biomed Pharmacother. 2020 Aug;128:110252. 10.1016/j.biopha.2020.110252. 32446112

[32] Milas O, Gadalean F, Vlad A, Dumitrascu V, Velciov S, Gluhovschi C, et al. Pro-inflammatory cytokines are associated with podocyte damage and proximal tubular dysfunction in the early stage of diabetic kidney disease in type 2 diabetes mellitus patients. J Diabetes Compl. 2020 Feb;34(2):107479. 10.1016/j.jdiacomp.2019.107479. 31806428

[33] Yang Z, Guo Z, Dong J, Sheng S, Wang Y, Yu L, et al. miR-374a Regulates inflammatory response in diabetic nephropathy by targeting MCP-1 Expression. Front Pharmacol. 2018 Aug 10. eCollection 2018;9:900. 10.3389/fphar.2018.00900. PMC6095963 30147653

[34] Singh B, Kumar A, Singh H, Kaur S, Arora S, Singh B. Protective effect of vanillic acid against diabetes and diabetic nephropathy by attenuating oxidative stress and upregulation of NF-κB, TNF-αand COX-2 proteins in rats. Phytother Res. 2022 Mar;36(3):1338–52. 10.1002/ptr.7392. 35088468

[35] Chen Y, Wang YJ, Zhao Y, Wang JC. Carbohydrate response element binding protein (ChREBP) modulates the inflammatory response of mesangial cells in response to glucose. Biosci Rep. 2018 Dec 7;38(6):BSR20180767. 10.1042/BSR20180767. PMC6435501 30420491

[36] Gong W, Chen C, Xiong F, Yang Z, Wang Y, Huang J, et al. CKIP-1 ameliorates high glucose-induced expression of fibronectin and intercellular cell adhesion molecule-1 by activating the Nrf2/ARE pathway in glomerular mesangial cells. Biochem Pharmacol. 2016 Sep 15;116:140–52. 10.1016/j.bcp.2016.07.019. 27481061

[37] Iwata Y, Furuichi K, Hashimoto S, Yokota K, Yasuda H, Sakai N, et al. Pro-inflammatory/Th1 gene expression shift in high glucose stimulated mesangial cells and tubular epithelial cells. Biochem Biophys Res Commun. 2014 Jan 17;443(3):969–74. 10.1016/j.bbrc.2013.12.072. 24361893

[38] Racchi M, Sinforiani E, Govoni S, Marinovich M, Galli CL, Corsini E. RACK-1 expression and cytokine production in leukocytes obtained from AD patients. Aging Clin Exp Res. 2006;18(2):153–7. 10.1007/BF03327432 16702786

[39] Yin H, Song S, Pan X. Correction to: Knockdown of miR-155 protects microglia against LPS-induced inflammatory injury via targeting RACK1: a novel research for intracranial infection. Aging Clin Exp Res. 2006 Apr;18(2):153–7. 10.1007/BF03327432. PMC5883406 29636642

[40] Li X, Xiao Y, Fan S, Xiao M, Wang X, Chen X, et al. RACK1 overexpression associates with pancreatic ductal adenocarcinoma growth and poor prognosis. Exp Mol Pathol. 2016 Oct;101(2):176–86. 10.1016/j.yexmp.2016.08.001. 27498047

[41] Schmid H, Boucherot A, Yasuda Y, Henger A, Brunner B, Eichinger F, et al. European Renal cDNA Bank (ERCB) C. Modular activation of nuclear factor-kappaB transcriptional programs in human diabetic nephropathy. Diabetes. 2006 Nov;55(11):2993–3003. 10.2337/db06-0477. 17065335

[42] Mezzano S, Aros C, Droguett A, Burgos ME, Ardiles L, Flores C, et al. NF-kappaB activation and overexpression of regulated genes in human diabetic nephropathy. Nephrol Dial Transpl. 2004 Oct;19(10):2505–12. 10.1093/ndt/gfh207. 15280531

[43] Wada J, Makino H. Inflammation and the pathogenesis of diabetic nephropathy. Clin Sci (Lond). 2013 Feb;124(3):139–52. 10.1042/CS20120198. 23075333

[44] Baker RG, Hayden MS. Ghosh S. NF-kappaB, inflammation, and metabolic disease. Cell Metab. 2011 Jan 5;13(1):11–22. 10.1016/j.cmet.2010.12.008. PMC3040418 21195345

[45] Dikmen K, Bostanci H, Gobut H, Yavuz A, Alper M, Kerem M. Recombinant adiponectin inhibits inflammation processes via NF-kB pathway in acute pancreatitis. Bratisl Lek Listy. 2018;119(10):619–24. 10.4149/BLL_2018_110. 30345768

[46] Nakano Y, Uchiyama M, Arima T, Nagasaka S, Igarashi T, Shimizu A, et al. PPARalpha agonist suppresses inflammation after Corneal Alkali burn by suppressing proinflammatory cytokines, MCP-1, and nuclear translocation of NF-kappaB. Molecules. 2018 Dec 29;24(1):114. 10.3390/molecules24010114. PMC6337747 30597991

